# The Excess Heat Factor: A Metric for Heatwave Intensity and Its Use in Classifying Heatwave Severity

**DOI:** 10.3390/ijerph120100227

**Published:** 2014-12-23

**Authors:** John R. Nairn, Robert J. B. Fawcett

**Affiliations:** 1 South Australian Regional Office, Bureau of Meteorology, Adelaide, South Australia 5067, Australia; 2 Bureau of Meteorology, Melbourne, Victoria 3008, Australia; E-Mail: r.fawcett@bom.gov.au; 3 Bushfire Cooperative Research Centre, East Melbourne, Victoria 3002, Australia

**Keywords:** heatwave, heatwave intensity, heatwave severity, excess heat factor, heatwave monitoring, heatwave forecasting, heat acclimatisation, heatwave adaptation

## Abstract

Heatwaves represent a significant natural hazard in Australia, arguably more hazardous to human life than bushfires, tropical cyclones and floods. In the 2008/2009 summer, for example, many more lives were lost to heatwaves than to that summer’s bushfires which were among the worst in the history of the Australian nation. For many years, these other forms of natural disaster have received much greater public attention than heatwaves, although there are some signs of change. We propose a new index, called the excess heat factor (EHF) for use in Australian heatwave monitoring and forecasting. The index is based on a three-day-averaged daily mean temperature (DMT), and is intended to capture heatwave intensity as it applies to human health outcomes, although its usefulness is likely to be much broader and with potential for international applicability. The index is described and placed in a climatological context in order to derive heatwave severity. Heatwave severity, as characterised by the climatological distribution of heatwave intensity, has been used to normalise the climatological variation in heatwave intensity range across Australia. This methodology was used to introduce a pilot national heatwave forecasting service for Australia during the 2013/2014 summer. Some results on the performance of the service are presented.

## 1. Introduction

Despite heatwaves being one of the most common natural hazards experienced across the Australian community, they remain imprecisely defined events with little understood varied impacts across different community sectors. The increasing availability of high-quality climate and weather-forecast temperature datasets offers an opportunity to build a shared understanding of the hazard posed by sequences of high temperature days.

Historically, heatwaves have been responsible for more deaths in Australia, Europe and the United States of America than any other natural hazard, including bushfires, storms, tropical cyclones and floods [1,2]. While heatwaves are not unusual for Australians, the trend towards more frequent and intense heatwaves [3,4,5] is of significant concern at home and abroad. McMichael *et al*. [6] has estimated that extreme temperatures currently contribute to the deaths of over 1,000 people aged over 65 each year across Australia. The number of heat-related deaths in temperate Australian cities is expected to rise considerably by 2050, as the frequency and intensity of heatwaves is projected to increase under climate change from global warming. Underpinning this view is the building evidence supporting the notion of a warming planet [7,8].

Heatwaves are frequently defined as a period of unusually or exceptionally hot weather. Extreme events typically occur in mid-summer, although severe and low-intense heatwaves are also experienced during spring and early autumn. We make a distinction between *heatwaves*, as periods which are hot in an absolute sense, and *warm spells*, as periods which are hot in a relative sense. Warm spells in this sense may occur at any time of the year, even in the middle of winter, whereas heatwaves as intended here are necessarily restricted to the summer half-year. In climate terms, heatwaves are associated with unusually high temperatures, warm spells with unusually high temperature anomalies. Both concepts (heatwaves and warm spells) are intrinsically meaningful, and deserve study, but they are clearly not the same thing.

Several other definitions of heatwaves have been proposed previously for use in Australia. One by Pezza *et al*. [9] requires that the maximum temperature be above the 90th percentile for three consecutive days, with the minimum temperature being also above the 90th percentile for the second and third days. If the 90th percentile thresholds are calculated with respect to the entire year, then heatwaves will be diagnosed, whereas if the percentile thresholds are relative to the calendar month or season, then warm spells will be diagnosed. In the former case, the heatwaves diagnosed by the Pezza *et al*. [9] method will have much in common with the heatwaves diagnosed by the EHF method proposed here. In the latter case, the warm spells diagnosed will have much in common with our heatwaves in the summer months, but less so during the rest of the year. Perkins and Alexander [10] have compared a wide range of warm spell and heatwave indices, noting the utility of differing indices dependent upon their intended use. In this regard warm spell indices provide information relevant to seasonally dependant temperature requirements in agriculture, whilst heatwave indices are relevant to timing adaptive measures when dealing with unusual temperature extremes.

Heatwaves in Australia are driven by slow-moving synoptic-scale events that allow the continuous development of hot air masses to persist over large areas for a period of days and in rare events, weeks. Fortunately, modern numerical weather prediction (NWP) models are quite good at forecasting such slow-moving systems and provide good guidance on the evolution of high temperature events on the one to seven-day time scale. As a consequence, heatwaves as a meteorological phenomenon are readily predicted by current operational standards.

Several recent studies [9,11,12,13,14,15,16] have looked at the climatic, synoptic and dynamic mechanisms responsible for causing intense heatwaves. Dry soils result in greater sensible heating of the lower atmosphere during the day through the reduction in evaporative cooling. Slow-moving deeply formed anticyclones recirculate deeply mixed hot boundary-layer air resulting in an environment that accrues excess heat. Additional dynamical links to tropical cyclone development at lower latitudes have also been shown to enhance the transport of heat from the upper tropical atmosphere to the boundary layer over Australia [11].

In Australia, heatwaves have traditionally been defined by the achievement of a minimum sequence of consecutive days where daily maximum temperatures reach a designated threshold. However, daily maximum temperatures are only part of the story when considering impacts on human health, agriculture, infrastructure, the demand on utilities (water, electricity, *etc.*) and other environmental hazards such as fire. Previous research has highlighted the importance of incorporating minimum temperature through the utilisation of daily mean temperature [17,18], a line of thought we follow here. The extent to which heat is dissipated overnight following a very hot day dictates the accumulating thermal load impacting vulnerable people and systems. The accumulation of this heat which is not being dissipated overnight results in “excess heat”.

Heatwave intensity occupies a continuum on which low-intensity heatwaves have little impact whilst more intense events inflict severe consequences upon the community and business sectors. Rising intensity leads to extreme outcomes where widespread adverse impacts are experienced. Impacts will vary according to each location’s experience or climatology of excess heat and each community’s capacity to develop resilient strategies. By measuring heatwaves within a scale that captures intensity, it becomes possible to differentiate between heatwave events. This in turn permits a sensible analysis of resilient strategies that can be usefully shared between communities learning to mitigate the escalating impact of increasingly intense heatwaves.

We propose a new index, called the excess heat factor (EHF), which is based on three-day-averaged daily mean temperature (DMT). This index is suitable for a nationally consistent heatwave service and could help inform emerging World Meteorological Organization (WMO) guidelines on the development of national heatwave/heat health services. A heatwave service utilising this measure of intensity would provide information to enable the Australian community to self-assess thresholds of vulnerability to periods of excess heat, and for the Bureau of Meteorology to forecast and warn when severe or extreme heatwaves threaten. Analysis and forecasts of low-intensity heatwaves would also be included in a heatwave service. Measurement and tracking of more frequent low-intensity heatwaves reinforces that the community possesses resilient adaptation strategies for sequences of normal hot summer days. Acknowledgement of the community’s inherent adaption to low-intensity heatwaves provides an opportunity for cultural acceptance that increasingly intense heatwaves are more hazardous and require adaptive and subsequent protective responses.

The choice of a three-day period (TDP) over which to calculate heatwave indices is motivated by studies of human responses to the onset of extremely hot weather. Epidemiological studies in Australia have identified health impact delays of between one day in Melbourne [18] and three days in Adelaide [19]. Adelaide’s mean summer (December, January and February) temperature is 3 °C higher than Melbourne resulting in a more resilient heat-adapted city capable of withstanding the impact of extreme heat for longer. This is consistent with lags of three and two days identified in Barcelona [20] and London [21] respectively. This is also illustrated in Nairn and Fawcett [22] (Figure 9 therein), in terms of heat-related mortality in South Australia during the 2009 heatwave, using data obtained from Langlois *et al*. [23]. In that event, it takes three days of very hot weather for the mortality rate to rise significantly above its antecedent rate.

Relative humidity can be an important consideration in assessing the human health effects of heatwaves. It is not observed and forecast as well as air temperature, however. On this basis we have chosen not to include it explicitly in our new heatwave metric. It does, however, have an implicit presence through our inclusion of daily minimum temperature. High humidity tends to result in high minimum temperature, and low humidity in low minima, and this will be reflected in our DMT calculation.

The heatwave literature has predominantly focussed on human health outcomes. Consequently sensible and latent heat are invariably combined together in order to account for effectiveness of thermo-regulation of biological systems. Frequently, regression equations [6,24,25,26,27] or synoptic air masses [28] are used to relate and measure impacts on human health outcomes at city or regional scales. At this level of interplay between multiple variables, units and outcomes it is difficult to visualise or compare heatwaves across time or compare the severity of local, national or international events. The use of heatwave indices that consider radiation balances at the human level such as PET (Physiologically Equivalent Temperature) [5] rely upon humidity data of variable quality.

Taking a step back from human impact, it is interesting to consider heatwaves as events where excessive sensible heat accumulates, resulting in a rising thermal load. Robinson [29] adopted a de facto heatwave definition based on heat watch and warning criteria developed by the US National Weather Service. Robinson’s approach incorporated frequency of exceedance of a fixed percentile of all observed heat index values [30,31,32]. Whilst an advance in developing an objective heatwave definition, heat index is difficult to employ in climate assessments and projections as past and projected records of humidity are difficult to create and quality control. Robinson’s work established a baseline climate description of heatwaves for the United States of America, but was not considered able to provide a complete time series of events nor be suitable for epidemiological purposes. Characterising and carrying out comparative investigations across heatwaves is desired.

The constituents of the EHF calculation (*i.e.*, daily maximum and minimum temperature data) have been reliably recorded and corrected in high-quality climate monitoring systems. Looking forward, surface temperature is projected with sufficient skill [33,34] in general circulation models (GCM), and indeed our new index has been used in climate studies [8,10] as a means of analysing heatwave trends in historical data. In consequence, the new index provides a new set of tools informing policy makers on global and Australian trends in heatwave frequency, intensity and distribution.

The new index supports an intensity and classification scheme which is relative to the local climate. Such an approach is clearly necessary given the abundant evidence that people are largely adapted to the local climate, in their physiology, culture and engineered supporting infrastructure [35]. The climate record is used to produce a significant heat intensity population sample suitable for classifying heatwaves by their level of severity. This is a subtle but significant shift from epidemiological studies that commence their investigation from the perspective of human population impacts. This allows our investigation to exploit the tools available to climate, weather prediction and climate projection science to develop a physical interpretation of heatwaves. This new perspective offers spatial and temporal coherence of heatwave intensity and severity by characterising heatwave intensity through a universal independent energy index. This allows for analysis and comparison of heatwave impact whilst considering the effectiveness of alternate mitigation strategies. The spatial evolution of heatwave intensity provides a new metric for assessment of impact. We can now investigate sensible heat impact before other contributors to human health impacts are considered.

Understanding the climatological recurrence of heatwaves across Australia’s diverse climatic regimes, from the tropical north to the near mid-latitudes of Tasmania in the south creates an understanding of Australia’s incidence of heatwave and differing levels of intensity. The ability to compare heatwave severity across jurisdictions, regions and cities provides an opportunity to compare resilience strategies and their relevance to other locations. This guidance has not been available to Australian policy makers previously, and provides a platform for development of mitigation strategies. The capacity to forecast the severity of heatwaves and monitor the regions affected provides intelligence that has not been available to the Australian community previously.

The structure of this paper is as follows: the new index is defined in Section 2. The datasets we have used in the construction of the index are presented in Section 3. Section 4 presents some basic climatological results for the index, with further discussion in Section 5, and an application of the index to a significant Australian heatwave is given in Section 6. Concluding remarks are given in Section 7. A separate paper currently in preparation will expand on this by illustrating the performance of the new index in relation to some notable Australian and international heatwaves. We note that the methodology described here is readily adapted to provide an analogous formulation for coldwave monitoring and prediction [22], but in this paper we restrict our attention to heatwaves.

**Figure 1:**
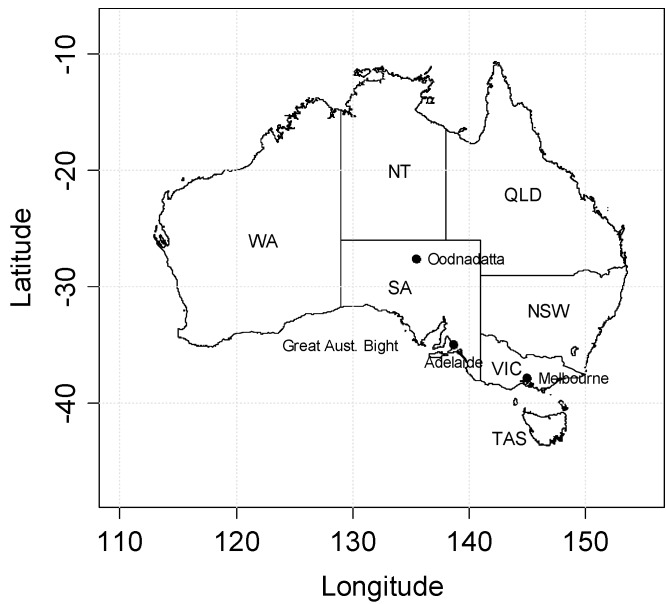
Map showing the Australian States/Territory and other locations mentioned in the text.

A pilot heatwave forecasting service for Australia based on the EHF was introduced in January 2014 for the latter part of the 2013/2014 Australian summer. We present in the Appendix some calculations on the performance of the forecasts across the summer. Subsequent consultation with State and Territory health and emergency sector stakeholders from across Australia found the service appropriately matched their requirements. Recommended service adjustments are under consideration for improved alignment across the sector’s mitigation and response plans. The Australian jurisdictions (State and Territory) and locations mentioned in the text are shown in Figure 1.

## 2. Methodology

The EHF is a new measure of heatwave intensity, incorporating two ingredients. The first ingredient is a measure of how hot a three-day period (TDP) is with respect to an annual temperature threshold at each particular location. If the daily mean temperature (DMT) averaged over the TDP is higher than the climatological 95th percentile for DMT (hereafter *T*_95_), then the TDP and each day within in it are deemed to be in heatwave conditions. On average, around 18 days per year will have a DMT exceeding *T*_95_, but it is necessary to have three high DMTs in succession in order to form a heatwave according to this characterisation. The second ingredient is a measure of how hot the TDP is with respect to the recent past (specifically the previous 30 days). This takes into account the idea that people acclimatise (at least to some extent) to their local climate, with respect to its temperature variation across latitude and throughout the year, but may not be prepared for a sudden rise in temperature above that of the recent past.

In Australia, daily maximum and minimum temperatures are measured in degrees Celsius (°C) and in relation to 24-h periods ending at 9 am local clock time (LCT), which means local standard time (LST) in those States/Territories which do not observe daylight saving time practices, and a combination of LST and local daylight time (LDT) in those States/Territories which do. In terms of the archiving of those daily temperatures, daily maximum (*minimum*) temperatures are archived for the 24 h from (*to*) 9 am LCT on the nominated day. This means that the daily maximum and minimum temperatures attributed to a particular calendar date typically (but not always) occur within the midnight-to-midnight calendar day, because the daily minimum is typically attained around sunrise and the daily maximum typically attained in the mid to late afternoon.

In terms of Australian historical data, daily maximum and minimum temperatures are available over long periods, but synoptic temperatures equally spaced throughout the day are not. Thus in Australia DMTs are typically calculated as the simple average of the daily maximum and daily minimum temperatures. There are consequently two possible choices for doing this calculation. The first choice has the daily minimum typically preceding the daily maximum, and because of the Australian data archiving conventions described above, this is the methodology normally used by the Bureau of Meteorology in its various climate monitoring activities, even though as far as the DMT is concerned the maximum and minimum temperatures used in the calculation actually occur in separate (adjacent) 9am-to-9am 24-h periods. The second choice, and the one adopted here, has the daily maximum typically preceding the daily minimum, and the two observations relate to the same 9am-to-9am 24-h period. We make this choice because of the human physiological response to a hot night following a hot day is more significant than the other way around [18].

Hence, let *T_i_* denote the DMT calculated in this way as the average of the maximum and the minimum which occur in the 24-h period from 9am LCT on day *i*. (In those parts of the world where there are equally spaced (around the clock) synoptic temperature observations extending back over many decades, it would be quite feasible to instead calculate the DMT using those synoptic observations, rather than the daily maximum and minimum temperatures, and that this approach might well be the preferred option where both options are available). Further, let *T*_95_ denote the 95th percentile of this DMT calculated across 1971–2000, using all days of the year in the calculation. Hence, on average *T_i_* will exceed *T*_95_ on around 18 days each year.

The two ingredients in the EHF calculation, as described above, are called excess heat indices (EHIs) and calculated as follows:

(1) $${EHI}_{sig}=(T_{i}+T_{i+1}+T_{i+2})/3\;–\;T_{95}$$

and:

(2) $${EHI}_{accl}=(T_{i}+T_{i+1}+T_{i+2})/3\;–\;(T_{i–1}+\;\ldots \;+T_{i–30})/30$$

In the first index, called the significance index, a three-day-averaged DMT is compared directly against the 95th percentile for DMT. If *EHI*_sig_ is positive, then the TDP is unusually warm with respect to the local annual climate. Conversely, if *EHI*_sig_ is negative or zero, then the TDP cannot be considered unusually hot, and so in order for a heatwave to be present we require *EHI*_sig_ to be positive. In terms of typical annual climates, this means that heatwaves according to this definition typically will not occur in the winter half-year.

In the second index, called the acclimatisation index, the same three-day-averaged DMT is compared against the average DMT over the recent past. Human physical adaptation to higher temperatures may take between two to six weeks [36], whilst engineered systems have a heat capacity design limit which frequently rely upon decision-support environmental precursors to apply adaptive measures to ensure reliable operation under higher temperatures. We have adopted the previous 30 days for this purpose. If *EHF*_accl_ is positive, then the three days are warmer (on average) than the recent past, and consequently there is now a lack of acclimatisation to the warmer temperatures and potential for adverse outcomes. Both of these EHIs can be thought of as temperature anomalies, the first with respect to the long-term climate, the second with respect to the recent past, and so both have temperature units (*i.e.*, °C).

We then propose to calculate our EHF as a product of these two indices, subject to the constraint that the EHF must have the same sign as the significance EHI. We do this via:

(3) $$EHF={EHI}_{sig}\times max(1,\;{EHI}_{accl})$$

with the units of EHF therefore being (°C)^2^, or alternatively and perhaps more conveniently K^2^. This formulation ensures that:

(4) $$sign(EHF)=sign({EHI}_{sig})$$

implying that a heatwave is present if EHF is positive (but not otherwise), but if additionally the acclimatisation EHI is positive, then that property amplifies its impact upon the EHF calculation. The duration of the heatwave comprises those days for which the significance index is positive, whether or not those days individually exceed *T*_95_ in their DMT. We note that it will be the case at the start and end of a heatwave for the EHF to be positive for one TDP and negative for an adjacent TDP (which overlaps the first by two days), with the potential for the overlapping days to be both in and not in heatwave. Accordingly we propose the classification rule mentioned above, that if a TDP has a positive EHF, then all the days within the TDP are considered to be heatwave days. Only if all three TDPs for which an individual day may fall have non-positive EHF do we consider the day to not be a heatwave day. By implication, an isolated hot day with DMT >*T*_95_ is not a sufficient condition for a heatwave.

In southern Australia, a heatwave will often end by the passage of a cold front and its associated rapid temperature drop. Thus some part of a TDP characterised as in heatwave conditions may not be hot, or even “cool” in terms of actual temperature, through being at the end of a heatwave, thus requiring some nuanced communication from the operational weather forecaster. Part of that communication will necessarily involve the fact that the DMT is falling or has fallen below *T*_95_. On the other hand, from the human impacts perspective, the fact that houses and other elements of the built environment may take several days following the cool change to cool down to pre-heatwave internal temperatures should not go unregarded.

The choice of the “1 °C” in equation 3 is somewhat arbitrary, at least for short heatwaves: essentially it is required to be small but positive. Negative EHF values signify the absence of a heatwave for that TDP, and we are not placing any interpretation at present on the magnitude of the negative values. Hence a re-specification in the form:

(5) $$EHF=max(0,\;{EHI}_{sig})\times max(1,\;{EHI}_{accl})$$

that is, a resetting of all negative values to zero, would not change the interpretation of the index as made in this paper.

During the spring months, TDPs with positive acclimatisation EHI should be relatively common (and analogously uncommon in the autumn months), but it is unlikely that the significance EHI will be simultaneously positive (except between November and March, as will be shown), hence the threshold for a heatwave would not be reached.

**Figure 2:**
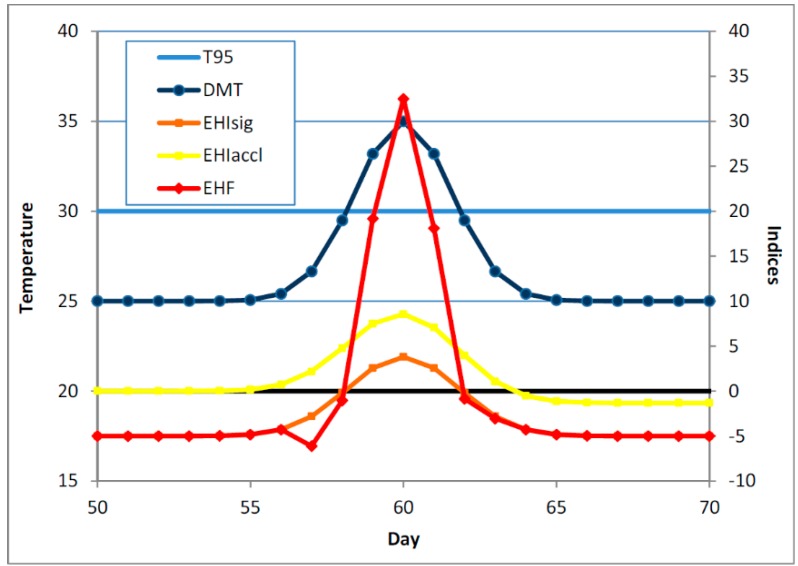
Schematic representation of a short heatwave early in the summer season. The DMT and 95th percentile thereof (both in °C) are plotted against the left hand axis, while the three heatwave indices (in °C and K^2^) are plotted against the right hand axis. The heatwave indices are plotted against the middle day of the TDP, to facilitate comparisons with the DMT profile. The zero line for the indices is shown as a thick black line. Because of the shortness of the heatwave, the acclimatisation EHI is positive for rather longer than is the significance EHI. The notional T_95_ value in the schematic is 30 °C.

**Figure 3:**
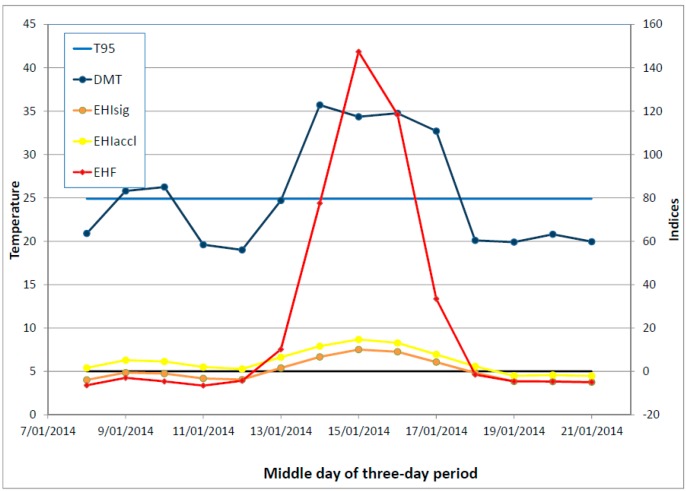
As per Figure 2 but for an actual short heatwave occurring in January 2014 in Melbourne, Australia. Data from the Melbourne Regional Office site (Bureau of Meteorology station number 086071). The *T*_95_ value at this site is 24.9 °C.

A short summer heatwave would typically occur within the context of a period of generally rising temperatures, so that the short period of positive significance EHI would occur within the context of a larger period of positive acclimatisation EHI. This is illustrated schematically in Figure 2, with an actual example shown in Figure 3. The DMT exceeds *T*_95_ for a short period (three days in the schematic example, four days in the actual example), which leads to the three-day-average DMT being above *T*_95_ for a likewise short period (comprising three overlapping TDPs in the schematic example, five overlapping TDPs in the actual example). The pattern of rising temperatures results in the acclimatisation EHI being positive for a much longer period, and provides a motivation for not allowing it to dictate the length of the heatwave (the three overlapping TDPs).

**Figure 4:**
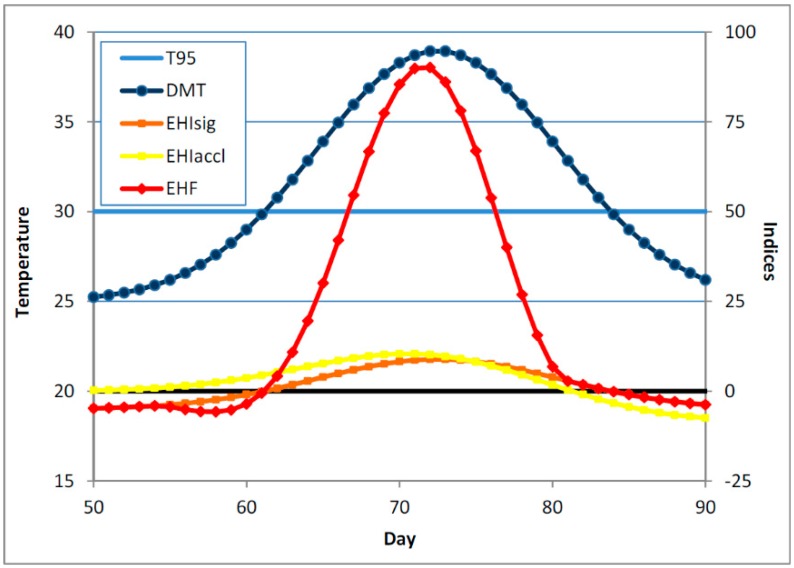
As per Figure 2, but for a long heatwave. Because of the length of the heatwave, the acclimatisation EHI can go negative before the end of the heatwave.

Figure 4 shows a schematic example of a much longer heatwave, one in which the period for which the DMT exceeds *T*_95_ is no longer short with respect to the acclimatisation window of 30 days. While the acclimatisation EHI is positive well before the significance EHI becomes positive (and the onset of the heatwave is deemed to have arrived), we can see that it is possible in a long heatwave for *EHI*_accl_ to go negative *before* the end of the heatwave, with the implication that because of the length of the heatwave there may be some acclimatisation or adaption occurring within the duration of the heatwave. This raises the difficulty of how to characterise the heat impact of a waning heatwave, where the DMT has started to fall, but not so much below *T*_95_ that the heatwave can be deemed to have ended. A consideration of this issue has influenced the form of our EHF definition, particularly the aspect of it where the *EHI*_accl_ only affects the magnitude of the EHF if it exceeds some minimum positive value. Accordingly, our previous statement about the “1 °C” in Equation (3) needs further elaboration: it should be small and positive, but not too small. A now-superseded construction of the EHF is given in Nairn *et al*. [37].

**Figure 5:**
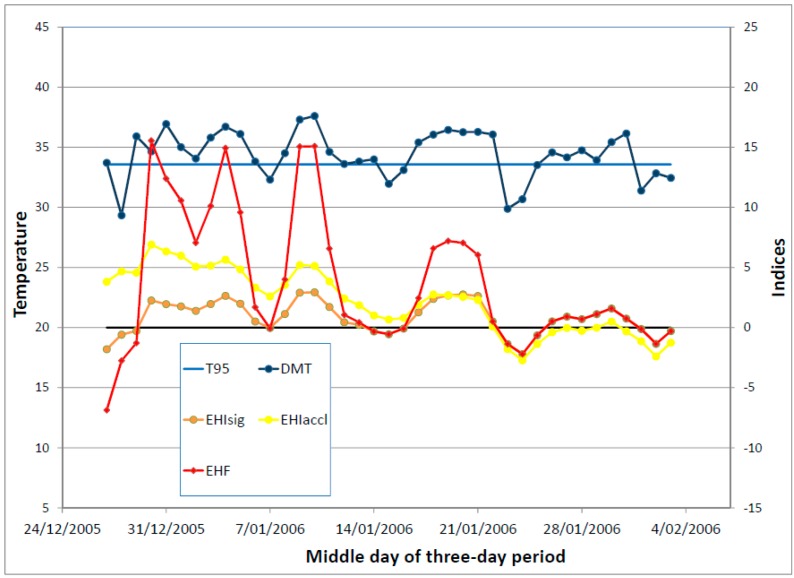
An actual period of extended heatwave activity at Oodnadatta (South Australia) in late 2005/early 2006. The DMT hovers around the heatwave threshold for the *EHI*_sig_ to exceed the *EHI*_accl_, and for *EHI*_accl_ to become negative while heatwave conditions are in place. Data are obtained from interpolated gridded analyses.

This issue, of *EHI*_accl_ becoming negative while heatwave conditions are still in place (*i.e.*, *EHI*_sig_ > 0), can also occur in the context of repeated shorter heatwaves, as illustrated in Figure 5. The data for Oodnadatta in inland Australia are obtained from gridded analyses (described in Section 3). They show an extended period of around six weeks where the DMT hovers around *T*_95_, causing repeated short heatwaves of low intensity. This episode is preceded by a period of cooler weather, and so *EHI*_sig_ < *EHI*_accl_ in the first half of the period represented in Figure 5, but by the end of the period the opposite is the case (*EHI*_sig_ > *EHI*_accl_), and indeed *EHI*_accl_ is negative at times while *EHI*_sig_ is still positive. The assumed acclimatisation to the protracted high temperatures is reflected in the declining amplitude of the EHF in Figure 5.

A threshold for severity is obtained at each location by counting all the TDPs within a climatology period (we have adopted 1958–2011 for this purpose), and computing the 85th percentile of all the positive EHF values within the climatology period, noting that the distribution of EHF is well described by the generalised Pareto distribution [22]. We denote this severity threshold *EHF*_85_. We will see that the severity threshold is far from being uniform across Australia, and that in fact there is a strong dependence of the severity threshold upon latitude. Hence it becomes useful to map the EHF for individual three-day heatwave periods as a multiple of the severity threshold. Lastly, we have chosen to designate a heatwave as being extreme if *EHF* ≥ 3 × *EHF*_85_.

The intent of these definitions is to create a heatwave intensity index and classification scheme which is relative to the local climate. Such an approach is clearly necessary given the abundant evidence that people and supporting infrastructure are largely adapted to the local climate, in physiology, culture and engineered supporting infrastructure.

## 3. Data

While excess heat indices may clearly be computed from site observational data, our principal dataset has been the 0.25°-resolution daily temperature analyses produced operationally by the Bureau of Meteorology [38]. These analyses are available back to 1911, but the underlying observational network is much sparser prior to 1957 in terms of its availability in digitised form. Therefore for most purposes, in particular climatological calculations, we only use the analyses from 1958 onwards. The analyses are near-whole-network analyses of site data that have been subjected to a considerable amount of quality control but no specific data homogenisation procedures.

These daily temperature analyses allow us to compute the EHIs and EHFs for all TDPs from 1958 onwards. Within the climatology period, statistics such as the mean positive EHF, the number of TDPs with positive EHF, and so on, may be calculated. The earlier data obviously may still be used to characterise particular heatwave events, in spite of the sparser observational network which lead us to exclude them from our climatological calculations.

## 4. Results

Climatologies of heatwave intensity and severity are described in this section. The location-specific heatwave methodology utilised establishes a baseline for the characteristics of heatwave severity across Australia.

**Figure 6:**
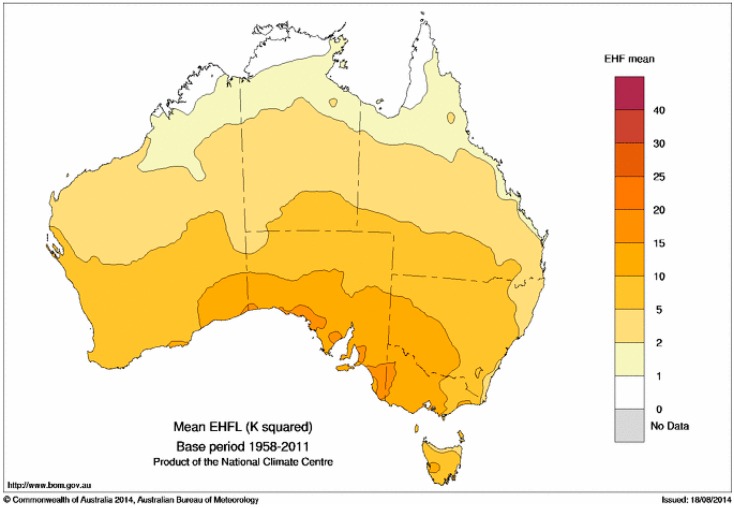
Mean positive EHF, in K^2^, based on all positive EHF values in the period 1958–2011, calculated using the gridded analyses of Jones *et al*. [38].

Figure 6 shows the mean positive EHF across Australia in the climatology period 1958–2011. Mean values are lowest in the tropical north, and highest around the southern continental coastline, resulting in a strong dependence of mean EHF upon latitude. This broadly reflects daily temperature variability.

**Figure 7:**
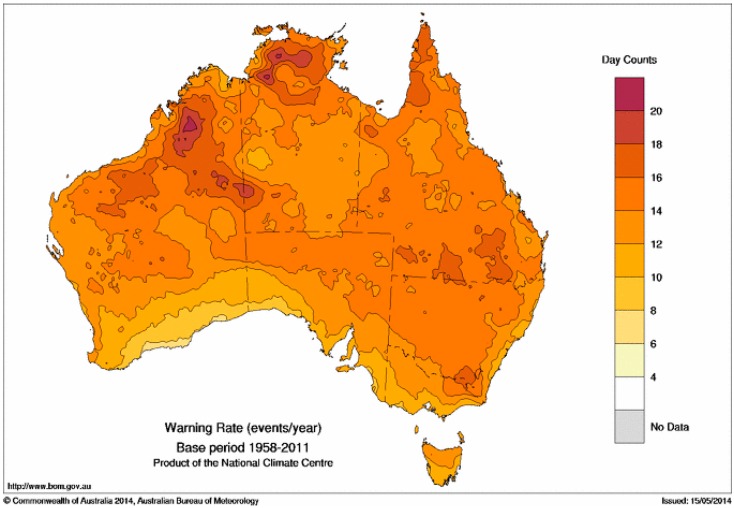
Average annual number of TDPs with positive EHF in the period 1958–2011.

Figure 7 shows the average annual number of TDPs with positive EHF across Australia in the period 1958–2011. The highest values are in the northwest and north, peaking at around 20 events per year. The lowest rates are in Tasmania and around the southern and southeast coasts of continental Australia.

**Figure 8:**
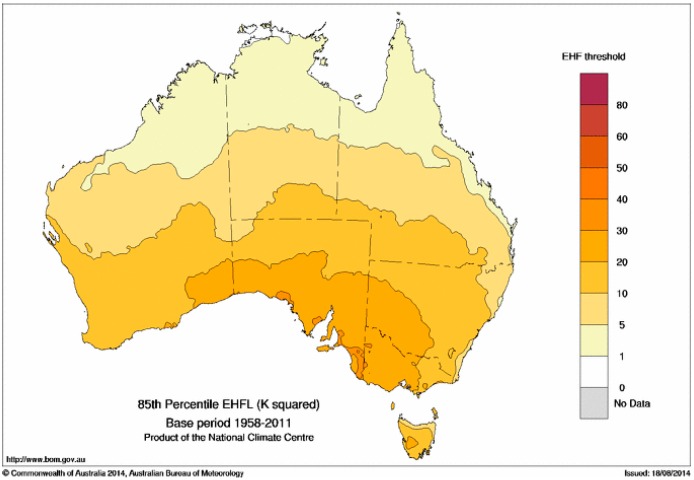
85th percentile of positive EHF values in the period 1958–2011 (in K^2^). These values are used as the threshold for a heatwave to be designated severe. The threshold for an extreme heatwave is taken to be three times the threshold for a severe heatwave.

The spatial pattern of the severity threshold *EHF*_85_ across this same period (Figure 8) is fairly similar to that of the mean positive EHF (Figure 6), and consequently there is a strong dependence of *EHF*_85_ upon latitude. Hence large temperature excursions are required in the south to cause a severe heatwave, according to the definition proposed here, while the corresponding temperature excursions required for the tropical north are much smaller. In consequence heatwave severity is likely to be more accurately predicted in the south, assuming that the ability to predict temperature itself (in terms of mean forecast errors) is approximately uniform across the country.

**Figure 9:**
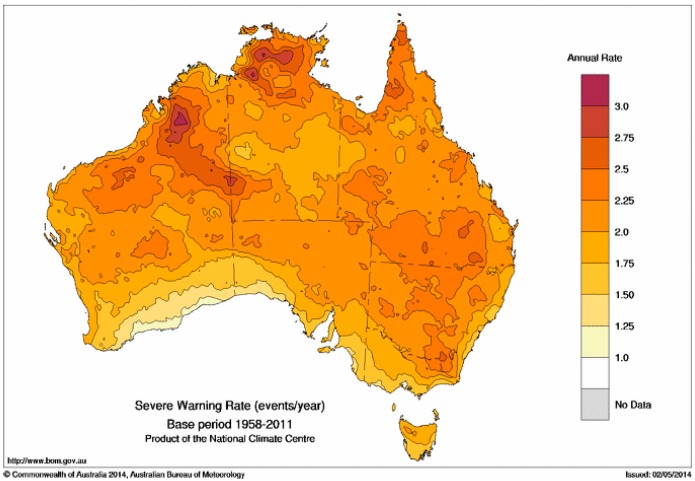
Average annual occurrence of TDPs with EHF above the severity threshold *EHF*_85_ in the period 1958–2011. Values are expressed in the form of TDPs per year.

Having chosen the severity threshold as shown in Figure 8, we calculate the average annual rate of TDPs with EHF exceeding the severity threshold. This calculation is shown in Figure 9, and shows a considerable degree of similarity to Figure 7. It is interesting that severe EHF TDPs occur more frequently in the tropical north, with the lowest rates being around the southern continental coastline, in spite of this being the region where the positive EHF values are typically largest. The occurrence rate for TDPs with positive EHF will be influenced by both the shape of the annual cycle and the short-range autocorrelation in DMT. A low short-range autocorrelation in DMT implies that a hot day is not likely to be followed by another hot day, thereby reducing the chance of a positive EHF and consequently the chance of a severe EHF.

**Figure 10:**
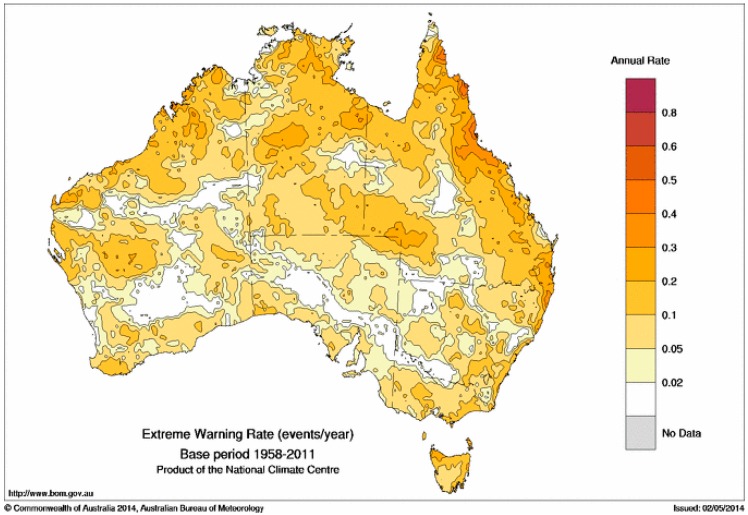
Average annual occurrence of TDPs with EHF above the extreme threshold in the period 1958–2011. Values are expressed in the form of TDPs per year.

An analogous calculation is done for the average annual occurrence of TDPs in the extreme range across the period 1958–2011 (Figure 10). Not surprisingly, extreme events occur much more infrequently than severe events at individual locations. The pattern in Figure 10 is also spatially much noisier than that shown in Figure 9, a statistical consequence of the rareness of these events.

**Figure 11:**
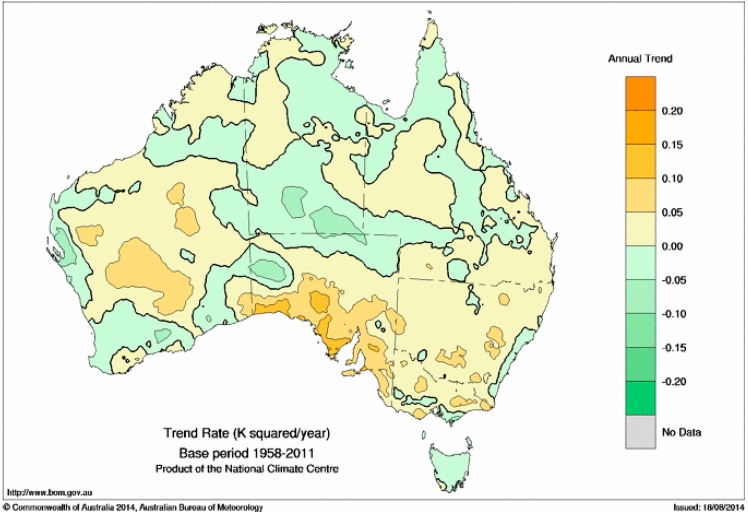
Trend in the intensity of EHF-positive events across 1958–2011. Values are expressed in units of K^2^ per year.

Figure 11 shows the linear trend in the intensity of EHF-positive events across the period 1958–2011. The trend is calculated in the usual way, using the ordinary least-squares (OLS) method, on points of the form (*t_i_*,EHF*_i_*) where *t_i_* represents the time variable and EHF*_i_* the corresponding EHF value, but only those points where the EHF value is positive are included in the calculation. The trends are positive across most of New South Wales and South Australia, but elsewhere in the country the spatial pattern is less consistent. The highest trends are around coastal South Australia, where they approach 0.15 K^2^/year. This implies an increase in the average intensity of heatwaves of up to 8 K^2^ across the study period. Not surprisingly, the strongest trends occur in the places of highest mean positive EHF (Figure 6).

We note as an important caveat to the results shown in Figure 11 that the residuals of the OLS linear regression calculation are a long way from being normally distributed. This is to be expected as the underlying data in the regression calculation consist of relatively many small positive EHF values and relatively few large positive EHF values. Accordingly we have also computed the linear trend using the Siegel [39] methodology, which should produce results which are much less sensitive to the presence of the long right tail of large positive EHF values. The Siegel linear trends (not shown) are considerably weaker than the OLS linear trends of Figure 11, suggesting that the OLS linear trends are being influenced to a considerable amount by the distribution (in time and amplitude) of the relatively infrequent but large positive EHF values, and so need to be interpreted with some caution.

**Figure 12:**
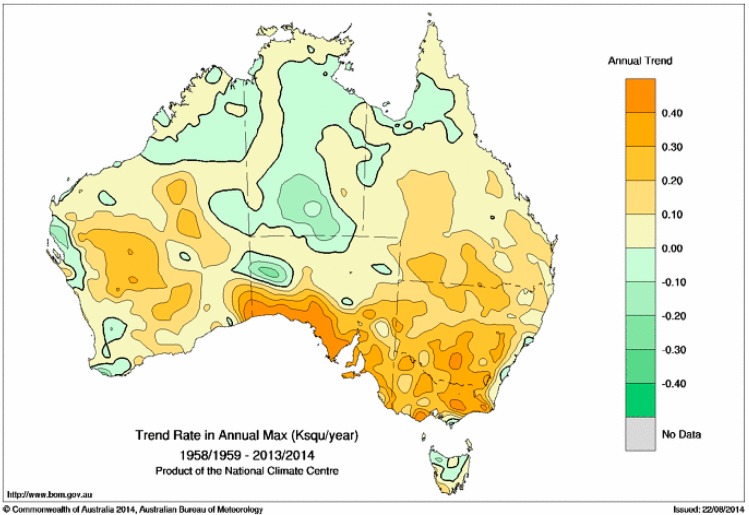
Trend in the annual maximum EHF across the period 1958/1959 to 2013/2014 (in K^2^/year).

**Figure 13:**
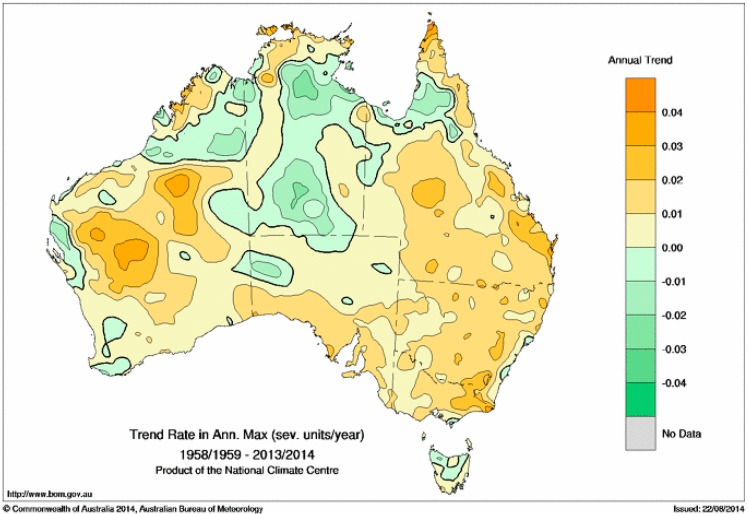
As per Figure 12 but in severity units per year.

This motivates our exploration of an alternative way of approaching the trend question is to calculate the annual maximum EHF value in each 12-month period, and then calculate the linear trend in those annual maxima. For the purposes of this calculation, we do this calculation over 12-month July-to-June periods, so that the summer period is in the middle of the 12 months. Figure 12 shows the trend in the annual maximum EHF, expressed in units of K^2^/year, while Figure 13 shows those trends in severity units per year. The calculation uses data from July 1958 to June 2014, and as before uses the (OLS) method.

Consistent with Figure 6 and Figure 8, the trend in the annual maximum is largest around the top of the Great Australian Bight, when expressed in units of K^2^/year. When the trend is expressed in severity units per year (Figure 13), we see that over a large part of eastern Australia, the annual maximum EHF has risen by around one half of a severity unit across the period represented by the calculation. Trends in the northern part of the country are more variable, with some negative trends seen. The stronger trend in the maximum heatwave intensity (Figure 12) compared to that of average heatwave intensity (Figure 11) suggests that heatwaves are becoming more intense. Heatwave extremes are rising faster.

The robustness of the trends shown in Figure 12, and consequently those shown in Figure 13, has been assessed by comparing the results of the OLS trend calculation with analogous calculations following the Sen [40] and Siegel [39] methodologies. The comparisons (not shown), while spatially noisier than the OLS calculation, suggest that the OLS calculation for Figure 12 is robust (unlike that for Figure 11).

## 5. Discussion

The distribution of mean EHF across Australia in Figure 6 reflects a narrower climatic temperature variation in the tropics during the warm season compared to southern Australia where northerly flow of hot air from the interior and cool changes sweeping in from the Southern Ocean generate a much wider temperature range. The same aspects of the synoptic climate lead to the 85th percentile of the positive EHF climate record (Figure 8) having similar characteristics. For this reason maps of EHF are difficult to interpret unless normalised to an impact or severity scale, something which we recommend doing.

Our interpretation of heatwave severity relies upon an expected local adaptation to low-intensity heatwaves which are frequently experienced, leading us to nominate the 85th percentile of all heatwaves in the climate record as a representative point at which we consider heatwaves to be no longer of low intensity. In earlier work, we found that heatwave intensities investigated for locations in Australia and elsewhere (including North America and Europe) are well modelled by a generalised Pareto distribution [22], and so the rapid rate of increase in intensity for the remaining 15% of heatwaves in the upper tail of the distribution is regarded as progressively more challenging for vulnerable people, requiring increasingly greater adaptive responses. For the last few percentage points of the heatwave population the remaining heatwave intensities are so extreme and rare that normally resilient people and engineered systems are vulnerable unless protective measures are adopted.

Historical Australian examples of extreme heatwaves occur chiefly between mid-December and late-February [22], coinciding with regional drought and longer days. The loss of evaporative cooling in dry soils and reduced radiative cooling due to shorter nights has been shown [41] to contribute to elevated minimum temperatures and higher levels of retained environmental heat during heatwaves.

Warning rates for low-intensity, severe and extreme heatwaves are shown in Figure 7, Figure 9 and Figure 10. The increased rate of warning in the tropics is likely to occur with seasonally drier soils prior to the arrival of warm-season rains. More intense heatwaves occur when warm-season rains are delayed with dry soils in combination with shorter nights contributing to higher minimum temperatures and more intense heat conditions. Extreme tropical heatwaves are most likely to occur when failed monsoon rains result in dry soils during January and February. Dry environments associated with extreme heatwaves present an interesting phase switch for northern (tropical) and eastern (sub-tropical) Australia, where low-intensity heatwaves occur in humid air masses. The transition from humid to dry conditions through the severe to extreme heatwave spectrum poses an interesting question. Adaptation strategies for humid heatwaves may not be appropriate for higher-intensity dry heatwaves. The spatial and temporal relationship between dry soils and more intense heatwaves will be explored in future investigations.

Southern Australian heatwaves away from the eastern sea board are normally dry, although occasional low-intensity heatwaves may be more humid according to the synoptic situation. As a consequence dry-atmosphere adaptation strategies are employed throughout the heatwave intensity range. The lower incidence rate for low-intensity and severe heatwaves (Figure 7 and Figure 8) over the southern coastal areas of the continent are counter-balanced by this strip experiencing a relatively higher extreme incidence rate (Figure 10). The episodic nature of heatwaves is more evident for this region. The rising trend in extreme heatwaves is evident for most of this area (Figure 12 and Figure 13) and large areas of eastern Australia, although the southwest of the continent has been experiencing a slight falling trend. This falling trend in the west may be a shift over time to synoptic conditions that permit more frequent coastal wind changes. Trends in heatwave patterns across Australia associated with trends in synoptic conditions will be explored in future investigations.

Australia’s heatwave climatology maps presented in Section 4 have set the stage for further heatwave discussion. It is now possible in Australia’s highly variable climate to examine the alternate antecedent conditions that result in differing rates of heatwave incidence and intensity.

## 6. Case Study: Southeast Australia 2009 Extreme Heatwave

**Figure 14:**
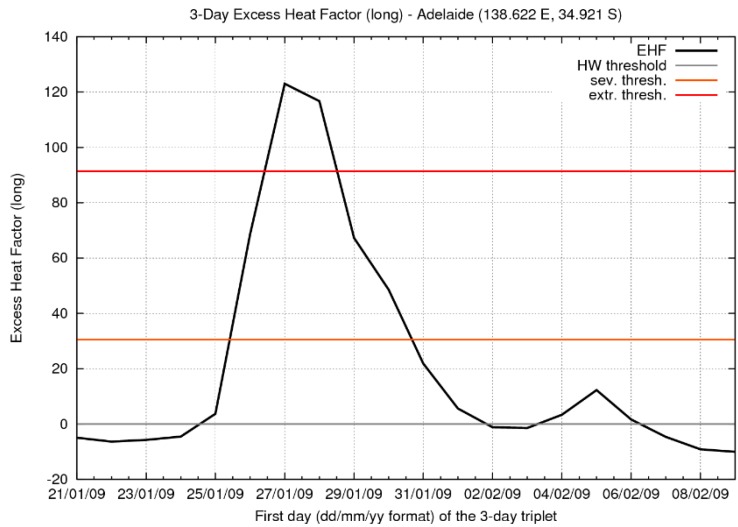
EHF for Adelaide (South Australia) across the period 21–23 January to 9–11 February 2009 (black line). The horizontal axis indicates the first day of each TDP. The horizontal grey line marks the threshold for a low-intensity heatwave (*i.e.*, zero EHF), while the orange and red horizontal lines mark the thresholds for severe and extreme heatwaves respectively. Data are derived from interpolating gridded analyses of EHF. *T*_95_ = 24.9 °C, with the severity threshold being 30.5 K^2^.

**Figure 15:**
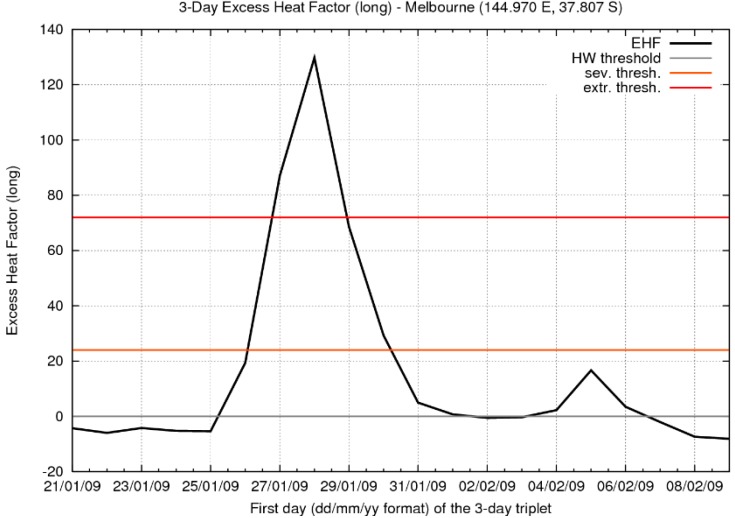
As per Figure 14 but for Melbourne (Victoria). *T*_95_ = 24.1 °C, with the severity threshold being 24.0 K^2^.

**Figure 16:**
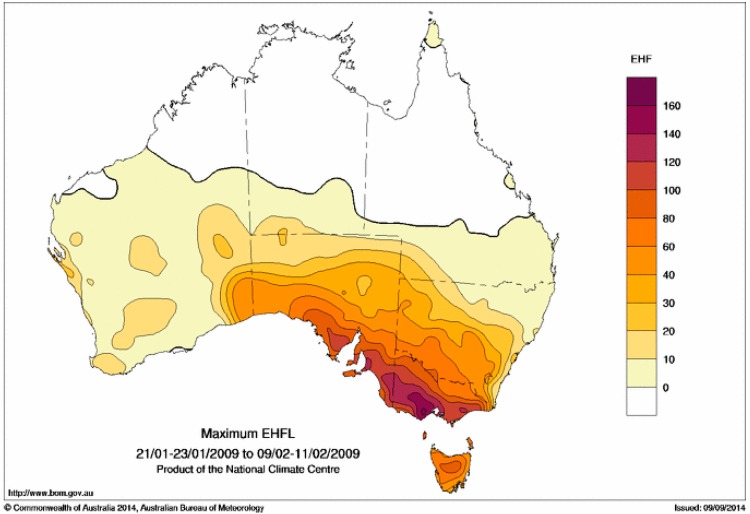
Maximum EHF for the period 21–23 January to 9–11 February 2009 (in K^2^).

**Figure 17:**
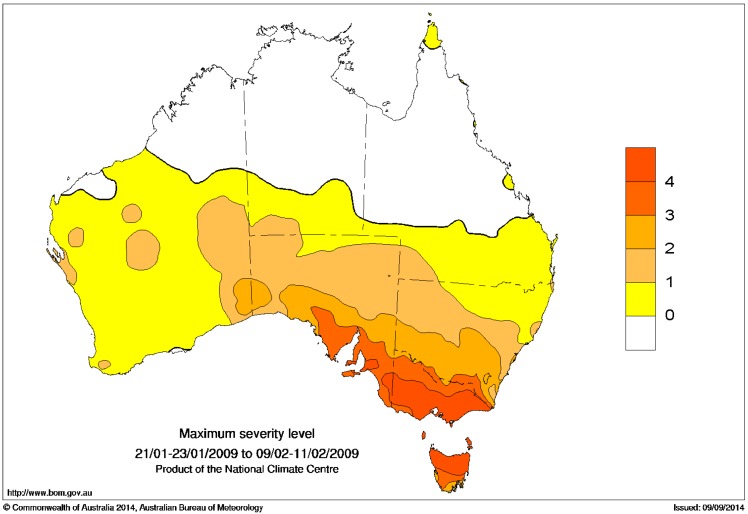
As per Figure 16 but expressed in multiples of the severity threshold. Yellow denotes a low-intensity heatwave (ratios between 0 and 1). Dark orange colours denote an extreme heatwave (ratios of 3 and higher). Ratios between 1 and 3 denote a severe but not extreme heatwave.

In this section we explore a significant heatwave which occurred across southeast Australia in January/February 2009 using the EHF and its associated metrics, noting that the graphical representations of the data shown in Figure 13, Figure 14, Figure 15, Figure 16 and Figure 17 could readily be adapted to a real-time weather forecasting context. At the end of January 2009, Adelaide (at the Kent Town site) saw five consecutive days with daily maximum temperatures above 41 °C (27–31 January), with the first four of them exceeding 43 °C. A maximum temperature of 40.6 °C on 1 February made six consecutive days above 40 °C. In consequence, the EHF exceeded the severity threshold in the Adelaide region by a factor of four (Figure 13) at the peak of the heatwave, placing the event well into the “extreme” range. Two further hot days (6–7 February) caused a minor resurgence of the heatwave index after the main event.

Melbourne (Victoria) saw three consecutive days with daily maximum temperatures above 43 °C (28–30 January) at the official weather site (Bureau Station Number 086071), and in the Melbourne area more generally the severity threshold was exceeded by a factor of more than five (Figure 15) at the peak of the heatwave. The resurgent heatwave was shorter in Melbourne than in Adelaide, effectively only lasting one TDP (ending 07 February), but that day saw the Melbourne official weather site’s hottest day on record (46.4 °C) and bushfires of appalling severity [42].

**Figure 18:**
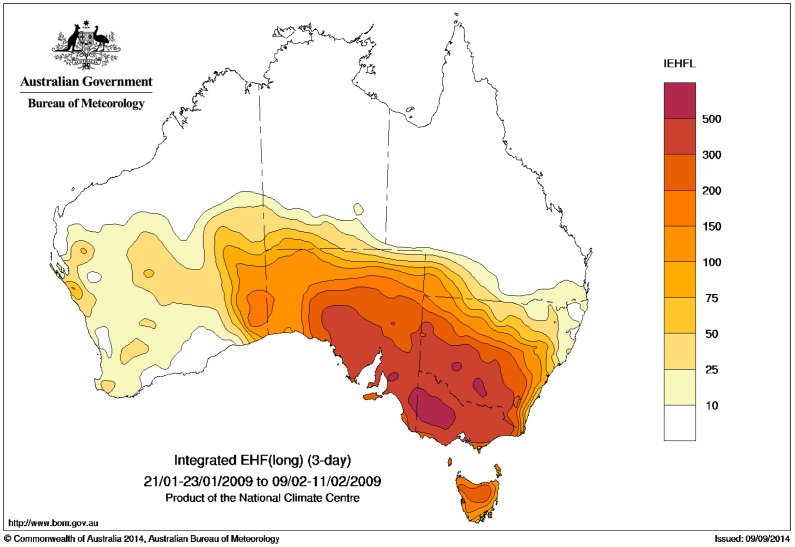
Integrated EHF across the period 21–23 January to 9–11 February 2009.

We present two different methods for ranking the scale of the heatwave. The first method is in terms of the maximum EHF value seen at each location within the heatwave period, to characterise the peak intensity. These maximum values at each location can be expressed either in actual values (Figure 16) or as multiples of the local severity threshold (Figure 17). The second method integrates or sums the positive EHF values across the heatwave period, to calculate the heat load of the entire event (Figure 18).

In terms of the integrated heat load (Figure 18), the heatwave extends across almost all of Victoria, southeast South Australia, southwestern New South Wales, and to a lesser extent northern Tasmania. The peak intensity in terms of actual EHF values (Figure 16) is highest in western Victoria, although in terms of severity (Figure 17) the heatwave reached “extreme” levels (ratios of three or higher) across most of Tasmania, almost all of Victoria and much of southeastern South Australia. Only parts of New South Wales close to the Victorian border experienced an “extreme” heatwave according to this metric. It should be noted though that much of Victoria and the northern half of Tasmania experienced particularly extreme conditions at the peak of the heatwave (as seen in the severe threshold multiples in Figure 17) where the severity threshold was exceeded by a factor of four.

Peak intensity and heat-load recorded for Adelaide (South Australia) and Melbourne (Victoria) in 2009 ranked amongst the top four heatwave events in their respective climate records. All of these events occurred at the end of significant multi-year droughts and were associated with significant bushfire outbreaks. Nairn and Fawcett [22] show how Adelaide’s peak intensity preceded the mortality peak by three days, with the intensity and mortality displaying similar characteristics. Ambulance heat-related tasks in Melbourne demonstrated a similar response.

Southeast Australia’s 2009 extreme heatwave resulted in South Australia recording 58 heat-related deaths [22,43] whilst Victoria reported 374 excess deaths [44]. By contrast the comparable 2003 extreme heatwave [22] in France recorded approximately 15,000 excess deaths [45]. The population ratio for France and Victoria is approximately 11:1 whilst the excess mortality ratio for these comparable extreme heat events is about 40:1. France’s approximate 4:1 excess mortality when compared to Victoria for these two extreme heatwave events provides context for comparison of resilience and adaptation measures employed during these events.

## 7. Concluding Remarks

A two-step process involving the calculation of heatwave intensity, and the normalisation of this intensity via a severity classification scheme has allowed an assessment of the spatial and temporal characteristics of low-intensity, severe and extreme heatwaves.

Heatwave intensity has been calculated as the product of the long-term and short-term daily mean temperature anomaly. Quality assured maximum and minimum temperature climate, forecast, seasonal and climate projection data present the opportunity to seamlessly assess how the intensity characteristics of heatwaves are changing for any location.

Impacts of past and future heatwaves across sectors with and without thermo-physiological vulnerability can be analysed coherently.

Whilst this heatwave intensity and severity percentile methodology has not involved humidity it has successfully categorised extreme heatwave events for both dry and humid climate regimes, where the highest heatwave impacts are observed across people, livestock, utilities, transport and economic activity. In Australia’s site-based daily temperature climate record (not shown) and in more recent, contemporary gridded climate and forecast data these high impact extreme heatwaves are found during periods of drought.

Future work will examine how severe and extreme heatwave classifications translate into levels of impact. In early studies it would appear that vulnerable populations are threatened as heatwaves become severe and that many more people and their supporting infrastructure are exposed as heatwaves become extreme. The value of identifying low-intensity heatwaves should also be emphasised. Most cultures value periods of lower-intensity heat, particularly if this comes as a shift in season from uncomfortably cool weather. Affirming cultural value for a level of heatwave that is not threatening to life provides a foothold for engaging and educating the public and business sectors in the dangers of more intense heatwaves.

The Appendix that follows demonstrates the performance of a heatwave service that has been piloted by the Australian Bureau of Meteorology utilising the heatwave intensity and severity methodology. The Bureau is also adapting the same methodology to sub-seasonal timescale as an experimental forecast product [46].

## Figures and Tables

**Figure A1:**
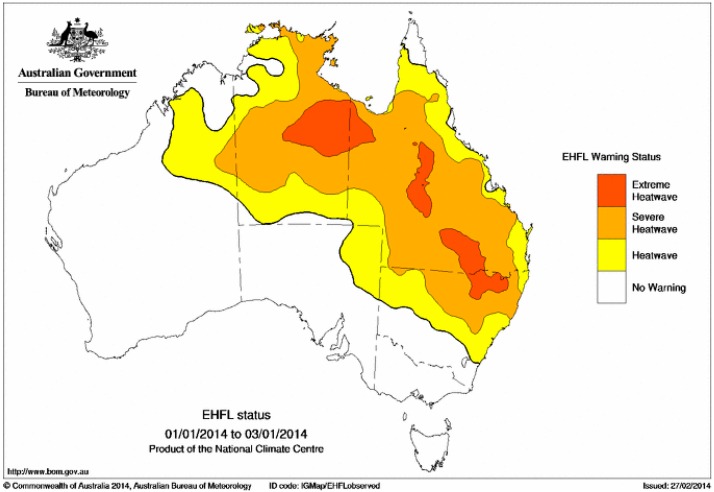
Heatwave observational analysis for the TDP 1 to 3 January 2014. The map shows the EHF expressed as a multiple of the severity threshold *EHF*_85_, thereby indicating four categories; no heatwave (white), non-severe or low-intensity heatwave (yellow), severe but not extreme heatwave (orange), and extreme heatwave (red). The forecasts were issued in largely the same format.

**Figure A2:**
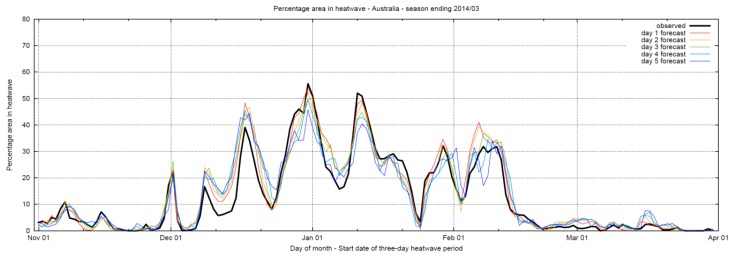
Percentage area of Australia in heatwave, as observed and forecast, across the period November 2013 to March 2014. The calculation is performed across continental Australia and the main island of Tasmania. Meridional convergence is taken into account when calculating the percentage areas.

**Figure A3:**
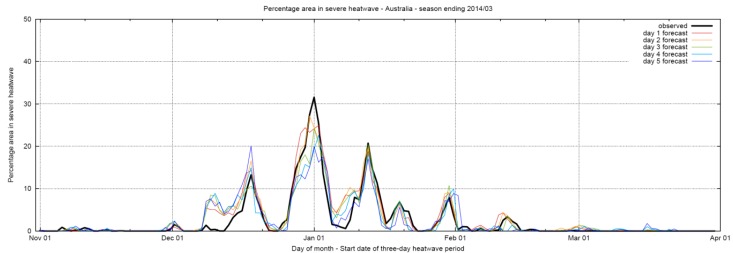
As per Figure A2 but for percentage area in severe heatwave.

**Figure A4:**
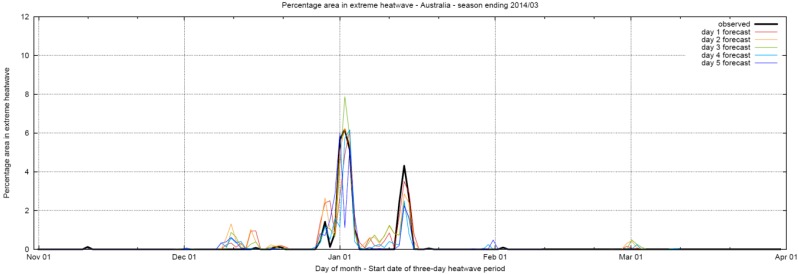
As per Figure A2 but for percentage area in extreme heatwave.

## Appendix

The Australian Bureau of Meteorology (the Bureau) utilised the EHF heatwave intensity and severity methodology to provide a pilot heatwave forecast service [47] for the summer of 2013/2014. Public distribution of the pilot products commenced on 8 January 2014, but the underlying forecasts were generated throughout the entire summer, and accordingly results for the period November 2013 to March 2014 are presented here. Daily maximum and minimum temperature forecasts were generated using the Bureau of Meteorology’s gridded optimal consensus forecasting system [48], allowing forecasts with lead times of around 12 (“day 1”), 36 (“day 2”), 60 (“day 3”), 84 (“day 4”) and 108 (“day 5”) hours between NWP model initialisation and the start of the TDP being forecast. This service provided images of heatwave severity with accompanying text for the next five TDPs. The forecasts are verified against EHF calculations derived from the Bureau’s operational daily temperature analyses [38]. An example of such a verifying analysis is shown in Figure A1. The forecasts were issued in largely the same format.

Figure A2 shows a comparison of the percentage area of Australia forecast and observed to be in heatwave during the 2013/2014 Australian summer. Figure A3 shows the corresponding results for severe heatwaves, and Figure A4 for extreme heatwaves. The comparison of the percentage areas gives a basic insight into whether the forecast system is over-forecasting or under-forecasting, although obviously it does not indicate if the forecasted heatwaves are in the correct places.

There was a considerable degree of heatwave activity during the summer, but two episodes were particularly outstanding. Those were in Queensland and the Northern Territory around the start of the New Year, and in southern Australia around two weeks later. The comparisons show that there is considerable skill in the ability to forecast non-severe and severe heatwaves, although perhaps less so for extreme heatwaves. There are some tendencies towards over-forecasting and under-forecasting, likewise some false alarms, but no significant events went unforecast.

The Bureau convened a national workshop on 30 April 2014 to review the performance of the pilot service. Invited stakeholders from health, emergency services, power utility and media sectors agreed to form a Heatwave Services Reference Group (HSRG). Feedback from this group has been incorporated into an updated heatwave forecast service for Australia’s 2014/2015 warm season which commenced during November 2014.

The Bureau is developing plans to upgrade the pilot heatwave forecasting service to the official forecast and warning system that supplies Australians with gridded forecasts and warnings that are quality controlled by forecasters and made available in a navigable, digital product suite that can be rendered for modern dissemination channels.
